# Mast Cells and Blood Vessels Profile in Oral Carcinogenesis: An Immunohistochemistry Study

**DOI:** 10.31557/APJCP.2020.21.4.1097

**Published:** 2020-04

**Authors:** Carolina Rodrigues Teófilo, Antonio Ernando Carlos Ferreira Junior, Aline Carvalho Batista, Francisco Vagnaldo Fechini Jamacaru, Fabricio Bitu Sousa, Mário Rogério Lima Mota, Malena Freitas e Silva, Paulo Goberlânio de Barros Silva, Ana Paula Negreiros Nunes Alves

**Affiliations:** 1 *Department of Dental Clinic, Division of Oral Pathology, Faculty of Pharmacy, Dentistry and Nursing, *; 3 *Department of Physiology and Pharmacology, Faculty of Medicine, Federal University of Ceará, Fortaleza, *; 2 *Oral Rehabilitation and Preventive Department, Dental School, Federal University of Goias, Goiana, Brazil. *

**Keywords:** Squamous cell carcinoma, mast cells, antigens, CD31, antigens, CD34

## Abstract

**Background:**

The objectives of the present study were to evaluate angiogenesis and mast cell density in oral epithelial dysplasia and oral squamous cell carcinoma (OSCC).

**Materials and Methods:**

This was an observational, retrospective and quantitative study. The samples consisted of 60 tissue specimens from patients with squamous cell carcinoma, epithelial dysplasia and controls (n=20/group). Immunohistochemistry was performed using an anti-tryptase antibody to mast cells and anti-CD31 and anti-CD34 for blood vessels and we count the number of mast cells and determine the percentage of CD31 and CD34 antibody staining (vascular density).

**Results:**

The mast cells had lower density in OSCC compared to control and dysplasia (p = 0.009). In angiogenesis, the expression of CD31 showed a higher percentage of blood vessels in OSCC (p < 0.001), however, CD34 showed no difference between groups (p=0.092). The CD31 antibody presented as a high immunostaining in oral mucosa than CD34.

**Conclusions:**

The increased vascularity in squamous cell carcinoma suggests that angiogenesis begins when malignant transformation starts that seems to be inversely associated with the number of mast cells.

## Introduction

Malignant neoplasms of the oral cavity are a serious public health problem in Brazil and worldwide. In the mouth, the most common histological type of malignant tumor is Oral Squamous Cell Carcinoma (OSCC), accounting for between 90-95% of the cases of oral cancer and this tumor is an invasive and aggressive epithelial neoplasm. The appearance of dysplastic lesions and the histological alterations that precede the development of malignant neoplasms often occurs. The main potentially malignant disorders (PMD) that affect the oral cavity are leukoplakia, erythroplakia and actinic cheilitis (Prado et al., 2010).

Angiogenesis is the emergence of new blood vessels from pre-existing capillaries. It is an essential step in tumor growth and metastasis because the new blood vessels provide oxygen and nutrition to the cells that are proliferating and facilitate the drainage of metabolites, in addition to functioning as a means of transportation for tumor cells (Souza et al., 2007). According to Folkman, cancer begins as avascular nodules (Folkman, 1971) and is only able to grow beyond 2 millimeters in three dimensions when the tumor cells become vascularized. 

Several cell types, such as tumor cells, endothelial cells, macrophages, and mast cells are involved in the increase in vascularization in neoplasms. Other factors that positively affect the signal for angiogenesis are protein action arising from oncogenes, hypoxia, low pH, poor nutrition and activity of reactive oxygen species (Viallard and Larrivée, 2017). Mast cells are directly involved in the evolution of neoplasia because, beyond their defense functions, mast cells participate in the regulation of the homeostasis of blood vessels (Alkhabuli, 2007). Their participation in this microenvironment has been suggested in various malignant tumors (Cimpean et al., 2017; Cherdantseva et al., 2017).

Mast cells are immune cells that originate in the bone marrow and migrate to peripheral tissues, where they undergo final maturation. They are long-term cells, residing primarily in connective tissue, most commonly near the epithelium. In histological sections, they are rounded or elongated with diameters between 8 and 20μ and have a large number of granules in their cytoplasm (Dvorak, 2005). The constitution of the mast cell granules is variable; they may produce and secrete a number of substances as chymase of basic fibroblast growth factor (bFGF), tryptase, heparin, histamine, tumor necrosis factor alpha (TNF-α), various interleukins (IL- 3, 4, 5, 6, 8, 10, 13,16), chemokines, matrix metalloproteinases (MMP-2 and 9), transformation growth factor beta (TGF-β), nerve growth factor (NGF), platelet-derived growth factor (PDGF) and vascular endothelial growth factor (VEGF) (Mukai et al., 2018; Theoharides et al., 2012).

Although the presence of mast cells around tumor masses has been recognized for more than 100 years, only recently has the attention of researchers turned more strongly to the role of this cell in neoplastic processes. In the oral cavity, there are few studies on mast cells reported in the literature and the results are conflicting (Iamaroon et al., 2003; Oliveira-Neto et al., 2007; Michailidou et al., 2008; Mohtasham et al., 2010; Sharma et al., 2010; Jahanshahi and Sabaghian, 2012; Kalra et al., 2012).

Few studies are devoted to studying angiogenesis in PMD. However, a gradual increase in micro vessel density, compared to normal tissue, has been described in premalignant lesions that originated in the uterus (Zyla et al., 2014), stomach (Feng et al. 2002), thyroid (Rajabi et al., 2019), and dysplastic nevi (Einspahr et al., 2007). Some studies of oral tumors have shown that the progression from normal tissue to epithelial dysplasia and squamous cell carcinoma (OSCC) is associated with increased vascularization (Orakash et al., 2018; Abbas et al., 2007).

As there are still conflicting results in the literature (Carla et al., 2012; Shivamallappa et al., 2011), the objective of this study was to evaluate angiogenesis and mast cell density in epithelial dysplasia and oral squamous cell carcinoma.

## Materials and Methods

*Study design*

This was an observational, retrospective and quantitative study in which 60 paraffin blocks for the assessment of angiogenesis and mast cells were selected from the archives of the Laboratory of Oral Pathology of the School of Dentistry, at the Federal University of Ceará, Brazil. The samples consisted of normal mucosa fragments, for use as controls, oral dysplasia and oral squamous cell carcinomas. Data related to the age and sex of patients as well as the location of the lesion were obtained from histopathological reports and the results are shown using descriptive statistics.

This study was submitted to the Ethics Committee of Federal University of Ceará and approved under protocol number 77/09.

*Immunohistochemistry*

Immunohistochemistry was performed on histological sections 5 µm thick on previously identified silanized histological slides, following the streptavidin-biotin-peroxidase technique. As primary antibodies, anti-tryptase (M7052, Dako^®^) diluted 1:1000 was used for the evaluation of mast cells, and anti-CD34 (M7165, Dako^®^) and anti-CD31 (M0823, Dako^®^), both 1:100 for evaluation of angiogenesis. The silanized histological slides were incubated at 70°C for 3 hours, and after this period, deparaffinized in xylene and alcohol gradient.

Antigen retrieval was performed in pH 9 buffer (S2367, DAKO Target Retrival), in Pascal pressure chamber (3:30 minutes 125°C, 18-24 psi). After blocking the endogenous peroxidase activity (aqueous solution of H_2_0_2_ 3%), the primary antibodies were incubated for 12 hours at 4°C in a humid chamber. After washing, the secondary biotinylated antibody was added (60 minutes), followed by streptavidin-biotin-peroxidase complex (60 min), and then revealed with chromogen diaminobenzidine (K3468, Dako^®^). The slides were counterstained with Harris hematoxylin for 30 seconds and mounted. As a negative control, the primary antibody was omitted from the reactions, and for a positive control, we used fragment mastocytoma, for anti-tryptase antibody, and pyogenic granuloma, for anti-CD31 and anti-CD34 antibodies.

*Capturing digital images*

Digital images of histological specimens were captured in a standardized way, using a light microscope (Olympus CX 31, Olympus^®^, Japan) equipped with a digital camera (Sony 10.1-megapixel, Sony^®^, Japan). The procedure consisted of an initial scan of the tumor, using a small magnification (40x) to identify areas of higher density. Then, using a magnification of 200x, color digital images were captured of the field with the higher concentration of vessels or mast cells. The images were stored in Windows^®^ Bitmap (BMP) format.

*Mast cell density*

With the software suited for microscopy (MBF Image J, MacBiophotonics, McMaster University, Hamilton, ON, Canada), brown stained cells that had mast cell compatible morphology were counted for the determination of the density of mast cells in each field.

*Percentage of vascular stain*

For the vascular quantification, Morphometric Analysis System (SAMM), a computer program developed specifically for this purpose, was used. The system was previously calibrated to recognize the color spectrum on the structure of interest (micro vessels), according to the technique used for staining. This procedure enables the software to automatically identify and target the blood vessels (separating them from the other components of the preparation). After the segmentation, the software determined the percentage of stained surface in the field. The calculation of area density was performed, which was defined by the ratio between the area occupied by microvasculature and the total area of the analyzed field.

*Statistical analysis*

The mast cell count and percentage of area stained by CD31 and CD34 antibodies were subjected to a Kolmogorov-Smirnov normality test and analyzed through the Kruskal-Wallis/Dunn (nonparametric data). All analyses were performed using Graph Pad Prism 5.0 (San Diego, California, EUA) software with a significance level of 95% for all assessments.

## Results

*Sample description*

The selected sample was composed of lesions present in individuals between 36 and 92 years old, with an average age of 64 years. Regarding gender, there was a slight predominance of males (50.3%). Samples of lip, palate, buccal mucosa, tongue, gingiva, floor and labial frenulum were collected.

*Mast cell density*

**Figure 1:**
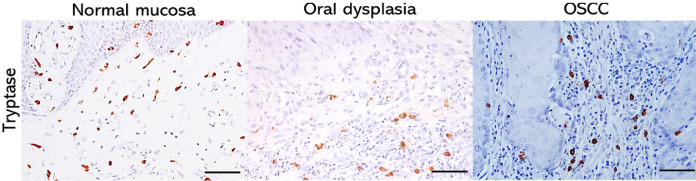
Photomicrography of Oral Mucosa Showing the Pattern of Tryptase Stain in OSCC, Epithelial Dysplasia and Normal Mucosa. Line = 50 µm (400x)

Mast cells were clearly identified as brown-colored, oval or elongated cells by immunohistochemical analysis using an anti-tryptase antibody (Figure 1). There was a statistically significant difference between the group of malignant lesions in relation to normal tissue and epithelial dysplasia (p=0.009), with OSCC having a lower concentration of mast cells than other lesions (Table 1).

*Vascular staining of vascular density (%)*

**Figure 2:**
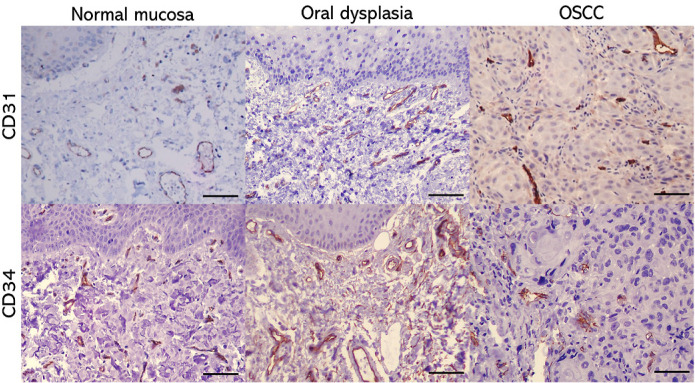
Photomicrography of Oral Mucosa Showing the Pattern of CD31 and CD34 Stain in OSCC, Epithelial Dysplasia and Normal Mucosa. Line = 50 µm (400x)

Blood vessels were identified as brown stained cells, solitary (considered vascular sprouts) or cell clusters with or without lumens by immunohistochemical analysis using anti-CD31 and anti-CD34 antibodies. There was more intense staining and higher background staining when the anti-CD34 antibody was used. The anti-CD31 stained poorly, but it was more specific for vascular endothelial sprouts and structures (Figure 2).

Statistically significant differences were found between the OSCC group and epithelial dysplasia and between OSCC and normal mucosa (p<0.001), but no differences were observed between normal mucosa and epithelial dysplasia. There were no statistically significant differences between groups (p = 0.922) (Table 1).

## Discussion

PMD are tissue alterations that have a high risk of progression to malignancy. It is of great importance to identify the occurrence of changes in the pattern of these lesions that indicate the progression of these to malignancy, enabling the development of agents that aim to halt this process and most of tumors of the oral cavity are preceded by PMD (Prado et al., 2010).

There is an increase in vascularization even before there is a tumor invasion during the formation of hyperplastic islets. However, some authors believe that premalignant lesions remain dormant before becoming angiogenic and proliferating (Shieh et al., 2004).

Several studies have been proposed to evaluate angiogenesis in premalignant lesions. In some, an increase in blood vessels is observed as the lesion progresses to malignancy, with fewer vessels observed in the normal epithelium, an increased number in epithelial dysplasia and an even higher prevalence in OSCC (Orakash et al., 2018; Iamaroon et al., 2003; Michailidou et al., 2008; Mohtasham et al., 2010; Gandolfo et al., 2011). Another study showed no difference between normal tissue and epithelial dysplasia, with significant difference only observed between OSCC and normal tissue, which corroborates the findings of this search (Shivamallappa et al., 2011). 

For quantification of vessels, a new method was used in the present study in which, after segmentation, the percentage of stains by the antibody was determined. This method allows a minor error in the results because the examiner variable was eliminated during the count. The literature provides several methodologies, in accordance to the number of reviewed fields and the magnification used for counting (Orakash et al., 2018; Carla et al., 2012; Shieh et al., 2004; Michailidou et al., 2008; Mohtasham et al., 2010; Shivamallappa et al., 2011). In this research paper, the field with the highest density in 200x magnification was selected. 

Currently, the most used markers for endothelial cells are the anti-CD31, anti-CD34, anti- VWF, anti- CD105 and VEGF antibodies. In our study anti-CD31 and anti-CD34 were used. The best method to assess angiogenesis in PML and oral cancer is currently under debate. Shieh et al. (2004) reported that CD31 and CD34 are better than factor VIII in oral OSCC and are often the markers of choice regarding tissues embedded in paraffin. In the present study, there was also no significant difference between normal mucosa, epithelial dysplasia and squamous cell carcinoma after staining for the anti-CD34 antibody. It was observed that, the CD34 marker stained structures other than vessels, mainly collagen fibers in the connective tissue, which may have contributed to the failure to obtain significant differences between groups.

Existing reports are controversial regarding the correlation between the density of mast cells and neoplasms (Gaje et al., 2016). There are reports of increased mast cells in malignancies compared to normal lung (Campillo-Navarro et al., 2014), and lymphomas (Duse et al., 2011). In such cases, it is believed that there is a role of mast cells in tumor progression via the release of mediators that promote angiogenesis. However, a recent study evaluating uterine malignancies found no statistically significant relationship between angiogenesis and mast cells, with no increase in the number of those with disease progression (Goksu Erol et al., 2011).

We found a decrease in the number of mast cells in the evolution of the lesion, with a significant difference between the groups of normal mucosa and epithelial dysplasia, and the OSCC group. These data are consistent with the results reported by Oliveira-Neto et al., (2007) and Kalra et al., (2012). However, others have shown an increase in mast cells in proportion to the increase in angiogenesis. In addition, previous studies showed that the density of mast cells increased as the aggressiveness of oral lesions progressed. Most likely, the varied biological behaviors of tumors, as well as the differentiated microenvironment, as determined by individual habits or the environment, explain these divergent results. It must be emphasized that the study of Oliveira-Neto et al., (2007), in which results similar to the present study were found, was also developed in a Brazilian population. Additionally, it is important to consider that different methodologies have been used to quantify mast cells, which may contribute to the conflicting results in the literature. 

Many substances are secreted by mast cells, which makes it difficult to determine the role of this cell in the cellular environment, especially in the presence of neoplasms. The production of agents that promote both the inhibition (IL-1, IL-4, IL-6, TNF-α) and the progression (VEGF, bFGF, tryptase, chymase, TGF- β, NGF e MMP 2 and 9) of tumors may have different effects on various tumors, depending on the local conditions of the stroma (Mukai et al., 2018).

It is well-known that most individuals presenting with oral cancer or premalignant changes make use of tobacco. A study that evaluated the effects of tobacco on oral mucosa showed that the carcinogen 4-nitroquinoline-N-oxide (4-NQO), present in tobacco, may cause a decrease in mast cells, which may explain the findings of this study (Sand et al., 2002).

Another consideration is that malignant cell proliferation preferentially occurs in places with low concentrations of mast cells because these cells may have anti-tumor activity and inhibit the growth of the neoplasm when present in high concentrations (Varricchi et al., 2017). Previous study concluded that mast cells have a direct inhibitory effect on the proliferation of cancer (Ekof and Nilsson, 2011). Furthermore, mast cells can recruit lymphocytes that act against aberrant cells through the release of IL-8 and RANTES, further hindering the growth of malignant neoplasms (Aoki et al., 2003).

The number of mast cells that migrate into the tissue can also be reduced, contributing to the low total number of mast cells in dysplasia and malignancies. Oliveira-Neto et al., (2007) observed this fact through the low number of c-kit positive mast cell and a low ratio between resident and recruited mast cells found in premalignant and malignant lesions of the oral mucosa. This is attributable to the release of low amounts of substances secreted by keratinocytes that attract mast cells and is also attributable to the oral tumor microenvironment. Parizi et al., (2010) performed a study comparing mast cell density (MD) in OSCC of skin and oral cavity, finding a lower MD in the latter, which is related to the reduced need for mast cell activation to promote increased vascularity in the oral mucosa.

Different techniques for the identification of mast cells in histological specimens are employed, ranging from histochemical reactions, such as those using toluidine blue (Parizi et al. 2010) and Alcian blue with Safranin (Campillo-Navarro et al., 2014), to reactions by immunohistochemistry monoclonal anti-tryptase (Mohtasham et al., 2010) antibody. Immunohistochemistry is the most specific technique for the detection of these cells (Shi and Lindholt, 2013) and the present study was performed with immunohistochemistry with anti-tryptase, 200x magnification, assessing the field with the highest density of mast cells.

Because mast cells are very complex cells and may take on different roles, both as a facilitator of growth and as an inhibitor of cancer and PMD, additional studies on their behavior in different tumor microenvironments are necessary. It is also important that there is standardization regarding the method used to assess both mast cells and angiogenesis, enabling a more reliable comparison of study results.

The CD-31 antibody was a better marker than anti-CD34 for the assessment of angiogenesis in oral mucosa. Increased vascularity in oral OSCC, when compared to the groups of epithelial dysplasia and normal mucosa, showed that angiogenesis is higher in tumors. However, the markers of angiogenesis were not effective for the detection of the increase of vessels in PMD.

In this research, a low density of mast cells in squamous cell carcinomas was observed. In our sample, there was no suggestion of involvement of mast cells in the angiogenesis process. However, further studies are needed to elucidate the hypothesis that increased vascularity in these tumors occurred through the release of pro-angiogenic factors by the tumor cells.

**Table 1:** Evaluation of Mast Cells and Vascular Density in Squamous Cells Carcinoma, Oral Dysplasia and Normal Mucosa

	Normal mucosa	Oral Dysplasia	OSCC	*P*-value
Mast Cells (number)	34.0±18.5	32.0±16.0	22.3±14.4*†	0.009
CD31 (vascular density)	3.3±1.9	4.5±3.4	9.6±5.5*†	<0.001
CD34 (vascular density)	8.9±1.3	10.0±6.6	8.2±3.8	0.092

*p<0.05 compared to the control group, †p<0.05 compared to OSCC; Kruskal-Wallis/Dunn (Mean±SD).
